# Excess Mortality in Istanbul during Extreme Heat Waves between 2013 and 2017

**DOI:** 10.3390/ijerph16224348

**Published:** 2019-11-07

**Authors:** Günay Can, Ümit Şahin, Uğurcan Sayılı, Marjolaine Dubé, Beril Kara, Hazal Cansu Acar, Barış İnan, Özden Aksu Sayman, Germain Lebel, Ray Bustinza, Hüseyin Küçükali, Umur Güven, Pierre Gosselin

**Affiliations:** 1 Department of Public Health, Cerrahpasa Faculty of Medicine, Istanbul University-Cerrahpasa, Kocamustafapasa, Fatih, 34098 Istanbul, Turkey; 2 Sabanci University Istanbul Policy Center, Bankalar Caddesi, No:2 Karaköy Minerva Han, 34420 Istanbul, Turkey; 3 Institut national de santé publique du Québec, 945 Avenue Wolfe, Québec, QC G1V 5B3, Canada; 4 Department of Public Health, Istanbul Medipol University School of Medicine, Kavacık mah. Ekinciler cad. No:19 Kavacık Kavşağı, Beykoz, 34810 Istanbul, Turkey; 5 Cerrahpasa Faculty of Medicine, Istanbul University-Cerrahpasa, Kocamustafapasa, Fatih, 34098 Istanbul, Turkey; 6 Institut national de santé publique du Québec and Université Laval, 945 Avenue Wolfe, Québec, QC G1V 5B3, Canada

**Keywords:** heat wave, mortality, cardiovascular, high temperature, Istanbul

## Abstract

Heat waves are one of the most common direct impacts of anthropogenic climate change and excess mortality their most apparent impact. While Turkey has experienced an increase in heat wave episodes between 1971 and 2016, no epidemiological studies have examined their potential impacts on public health so far. In this study excess mortality in Istanbul attributable to extreme heat wave episodes between 2013 and 2017 is presented. Total excess deaths were calculated using mortality rates across different categories, including age, sex, and cause of death. The analysis shows that three extreme heat waves in the summer months of 2015, 2016, and 2017, which covered 14 days in total, significantly increased the mortality rate and caused 419 excess deaths in 23 days of exposure. As climate simulations show that Turkey is one of the most vulnerable countries in the Europe region to the increased intensity of heat waves until the end of the 21st century, further studies about increased mortality and morbidity risks due to heat waves in Istanbul and other cities, as well as intervention studies, are necessary.

## 1. Introduction

Heat waves are one of the most common direct impacts of anthropogenic climate change. Frequency, intensity, and duration of warm spells and heat waves have increased due to global temperature rise [1]. The number and intensity of heat waves in the Mediterranean region, including western Turkey and the Black Sea coastline, have significantly increased as well [2,3]. Excess mortality due to heat wave episodes has been documented after several long and widespread heat wave episodes over the last 30 years, for example: the 1995 heat wave in the upper midwestern US, with more than 700 excess deaths in Chicago only [4]; the 2003 European heat wave episode with more than 70,000 total excess deaths in 16 European countries [5]; and the 2010 heat wave in India with 1344 excess deaths in the city of Ahmadabad alone [6].

Scientific research, local preparedness, and public health interventions clearly reduce the risk of mortality during heat waves. In France, for example, where a severe 2006 heat wave caused about 2065 excess deaths, the model predictions for expected mortality were much higher [7].

The number of hot days and frequency and duration of heat waves increased in the western part of Turkey between 1965 and 2006 [8]. The total number of heat wave episodes increased all over Turkey between 1971 and 2016, according to an analysis using the data of meteorological stations [9]. The heat waves in 2000, 2007, and 2010 in Turkey were investigated from a meteorological perspective [10], and there were several news reports regarding intensive heat waves and health-related deaths in southeastern Europe, including Turkey [11,12,13]. Temperature rise and increasing inter-annual temperature variability, especially during the summer seasons in the Mediterranean Basin until the end of the century, are evident from climate models simulations, and therefore, the quantity and intensity of the heat waves are expected to increase in Turkey due to global anthropogenic climate change [14].

However, there are very few studies on mortality related to heat waves available in Turkey. A study on the effects of heat on mortality in European and Eastern–Southern Mediterranean cities including Istanbul shows that a 1 °C increase above threshold causes a 2.4% increase in mortality on all ages [15]. Oray et al. [16] reported in an observational study that an increase in mortality was documented in an emergency department in Izmir during a nine-day period when the air temperature values were higher than the seasonal average of the city in June 2016. Heat-related mortality studies based on health statistics have been very limited, although Turkey has been affected by many well-studied Euro-Mediterranean heat waves, including one in the summer of 2003. Turkey is one of the most vulnerable countries to the increased intensity of heat waves until the end of the 21st century. Amengual et al. states that “By 2075–2094, projected extreme heat wave amplitude increases range from 2 °C to 4 °C per heat wave day in southern Spain, ... Italy, Greece, and Turkey...” [17].

In this context of increasing meteorological risks, a description of current impacts of heat waves is a first necessary step to begin addressing this public health risk now and in the future. Most existing research in this domain is North American and European, while the higher risks are probably in countries where such studies remain to be performed; however, they often are difficult to implement as detailed and high quality data availability remains a serious problem [18]. In this research, excess mortality in Istanbul attributable to extreme heat wave episodes between 2013 and 2017 is examined using total excess deaths in general and using mortality rates across different categories, including age, sex, and cause of death, in order to begin identify potential subgroups for preventative public health actions.

## 2. Methods

### 2.1. Study Area

Istanbul is the most populated city in Turkey, with 15 million inhabitants representing 18.5% of Turkey’s total population, according to the 2017 census [19]. The city is located in the northwestern part of the country at 41°01′ N, 28°58′ E, divided between the European and Asian continents by the Bosporus Strait. Despite its large population, the city covers only 5461 square kilometers; thus, its population density is high at 2747 inhabitants per km^2^. The southern half of the city, stretched along the coasts of the Bosporus Strait and Marmara Sea, is where most of the urban settlements and commercial areas are located. The climate in the city’s southern settlements has the general characteristics of the Mediterranean type climate, characterized by mostly warm and dry days in the summer and cold and wet days in the winter [20]. The mean annual temperature of the city is 13.8 °C, with the highest monthly average in August of 22.8 °C and the lowest monthly average in February of 5.3 °C. The warming trend in Istanbul is clear, as the observed mean annual temperature increased 0.94 °C between 1912 and 2016 [21]. Recently, however, settlements in the city have started to expand northward into rural and forested areas where the climate is modified by the cooler Black Sea patterns, which bring colder and rainier weather than a typical Mediterranean climate both for winter and summer seasons [20].

### 2.2. Mortality Data

Daily mortality records for the three summer months (June–August) every year from 2013 to 2017 were obtained from the Istanbul Health Directorate with their permission. The data starting from 2013 only was used because reliable information on the daily number of deaths in Istanbul was only available from this year. It included the deaths of Istanbul residents of all ages, while abortions and in-utero deaths were excluded, although reported by the same Death Notification System. The daily number of deaths was analyzed according to sex, age, and cause of death. The age at death was categorized into four groups: 0–14, 15–64, 65–74, and ≥75. The 75+ is a separate age group in order to see the vulnerability of elderly people. The cause of death was coded according to the International Classification of Diseases, Revision 10 (ICD 10). All non-accidental and non-violent deaths were classified as deaths due to cardiovascular diseases (I10–I99), respiratory diseases (J00–J99), or others. Crude, sex-specific, age-specific, and cause-specific mortality rates were calculated using the population data, which was obtained from the Turkish Statistical Institute, in order to estimate the excess death caused by extreme heat wave events.

### 2.3. Meteorological Data

The meteorological data used for this study included daily mean, maximum, and minimum temperatures for the summer months (June–August) of 2013–2017. The First Region Directorate (Istanbul) of the Turkish State Meteorological Service provided the meteorological data collected from 24 weather stations in the metropolitan city of Istanbul. The average of the mean daily temperatures of all 24 stations was used for the analysis, since the death records were collected for all districts of Istanbul in total, regardless of the place of death.

### 2.4. Definition of Heat Wave

Although there is no universal definition of a heat wave, periods of extreme temperatures in which the average daily temperature is greater than the 90th or 95th percentile for the region over a period of two or more days are usually considered as heat wave conditions [22,23]. In this study, an extreme heat wave episode was defined as a period with a daily average of the mean temperature above the 95th percentile for at least three consecutive days. Three days (lag days) were added at the end of the defined heat wave period in order to take into account the delayed impact of heat waves on mortality [24]. The reference period used for comparison was determined using the same days of the nearest week corresponding with the defined extreme heat wave in other years. If these dates coincided with any other heat wave episode, these days were excluded and these reference periods had 3 or 4 years, depending on relevant available data.

### 2.5. Statistical Analysis

SPSS v21.0 (SPSS Inc., Chicago, IL, USA) and Microsoft Office Excel (Microsoft Corporation, Redmond, WA, USA) were used for statistical analysis. Crude, sex-specific, age-specific, and cause-specific death rates were calculated by dividing the number of deaths over the multiplication of the population with the number of days. The daily excess number of deaths were thus calculated as the difference in death rates during the heat wave (including the lag days) and the comparison period, multiplied by the total population during the heat wave. Z tests of the difference of natural logarithms were used to compare death rates during the heat wave (including three added lag days) to the death rates during the reference period.

(1) $$Z=\frac{\ln\left(T_{heat\;wave}\right)-\ln\left(T_{reference\;period}\right)}{\sqrt{\frac{var\left(T_{heat\;wave}\right)}{{\left(T_{heat\;wave}\right)}^{2}}+\frac{var\left(T_{reference\;period}\right)}{{\left(T_{reference\;period}\right)}^{2}}}},$$

where

(2) var(T)=m(PJ)2,

where *T_heat wave_* is the crude daily death rate for a heat wave; *T_reference period_* is the crude daily death rate for the reference period; *m* is the number of deaths during the period; and *PJ* is the number of persons-days at risk during the period.

Normal approximation was used to calculate the *p*-value. Risk ratio (RR) was calculated by dividing the death rate in the heat wave by the death rate in the reference period, and the CI_95%_ was calculated. A *p*-value < 0.05 was accepted for statistical significance.

## 3. Results

For summer months, the average daily number of deaths in Istanbul hovered between 140 and 161 from 2013 to 2017. Depending on the year, the average of summer months daily mean temperatures was between 23.4 °C and 24.2 °C. Annual population numbers, daily average number of deaths according to sex and age group, and the daily minimum and maximum average temperatures are shown in Table 1. 

Four extreme heat wave episodes were identified between 2013 and 2017, based on the definition above; one heat wave happened in 2015, one in 2016, and two in 2017. There were no extreme heat wave episodes in 2013 and 2014. The dates and average of daily mean temperatures of the heat waves and their reference periods, crude death rates in each heat wave episode, and the risk ratios (RR) compared to the crude death rates in reference periods for each heat wave episode are shown in Table 2. 

The first heat wave (Heat Wave 1) started on 27 July 2015 and lasted four days, and the three-day lag period ended on 2 August 2015. Risk ratio for all deaths during Heat Wave 1 was 1.11 (CI_95%_ 1.04–1.18) and significantly increased (*p* = 0.002). This ratio corresponded with 17 daily and 118 total excess deaths in Heat Wave 1 in 2015.

The second heat wave (Heat Wave 2) started on 5 August 2016 and lasted seven days, and the three-day lag period ended on 14 August 2016. Risk ratio for all deaths during Heat Wave 2 was 1.06 (CI_95%_ 1.00–1.12) and significantly increased (*p* = 0.037). This ratio corresponded with 10 daily and 96 total excess deaths in Heat Wave 2 in 2016.

The third heat wave (Heat Wave 3) started on 29 June 2017 and lasted three days, and the three-day lag period ended on 4 July 2017. Risk ratio for all deaths during Heat Wave 3 was 1.21 (CI_95%_ 1.14–1.30) and significantly increased (*p* < 0.001). This ratio corresponded with 34 daily and 205 total excess deaths in Heat Wave 3 in 2017.

The fourth heat wave (Heat Wave 4) started on 5 August 2017 and lasted three days, and the three-day lag period ended on 10 August 2017. Risk ratio for all deaths during Heat Wave 4 was 0.99 (CI_95%_ 0.92–1.07) but was not significantly increased unlike the other three heat wave episodes.

**Figure 1:**
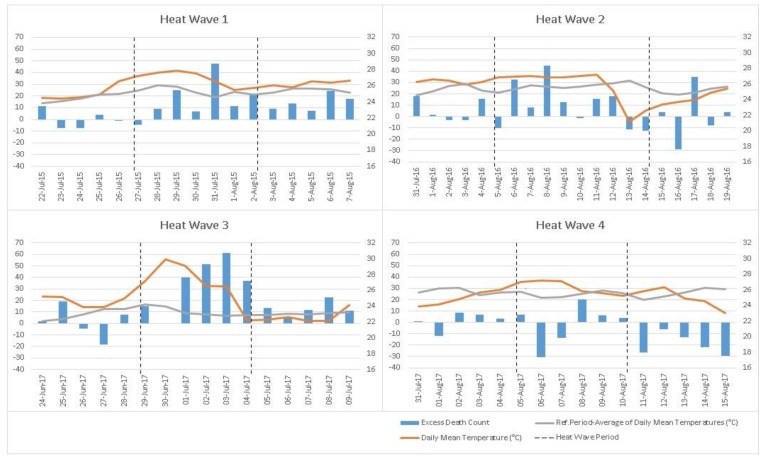
Excess death counts, daily mean temperatures, and temperature of reference periods for the Extreme Heat Waves between 2013 and 2017 (27 Jul –2 Aug 2015, 5 Aug–14 Aug 2016, 29 Jun–4 Jul 2017, 5 Aug–10 Aug 2017 respectively).

The analysis shows that three extreme heat waves in the summer months of 2015, 2016, and the first heat wave in 2017, which covered 14 days in total, significantly increased the mortality rate and caused 419 excess deaths in 23 days, including the three-day lags. Excess death counts, daily mean temperatures, and temperature of reference periods are shown for each heat wave in Figure 1.

Sex-specific death rates and risk ratios for men and women in the extreme heat waves between 2013 and 2017 are shown in Table 3. The death risk for men increased only in Heat Wave 3 in 2017 (RR: 1.14 (CI_95%_ 1.04–1.26)). The death risk for women, on the other hand, increased in Heat Wave 1 in 2015 (RR: 1.25 (CI_95%_ 1.15–1.37)) and Heat Wave 3 in 2016 (RR: 1.29 (CI_95%_ 1.18–1.42)). Neither sex-specific death risks were significantly increased during Heat Wave 2 in 2016.

Age-specific death rates and risk ratios for the age groups 0–14 and above 75 are shown in Table 4. While the death risk did not increase for the 0–14, 15–64, and 65–74 age groups (data not presented here), there was an increased risk for the ≥75 age group in Heat Wave 1 in 2015 (RR: 1.16 (CI_95%_ 1.06–1.27)) and Heat Wave 3 in 2017 (RR: 1.38 (CI_95%_ 1.26–1.52)).

Cause-specific death rates and risk ratios for cardiovascular, respiratory, and others are shown in Table 5. While there was no increased death risk for respiratory diseases, death risk caused by cardiovascular diseases significantly increased in Heat Wave 1 in 2015 (RR: 1.17 (CI_95%_ 1.05–1.31)) and Heat Wave 3 in 2017 (RR: 1.32 (CI_95%_ 1.18–1.48)).

**Figure 2:**
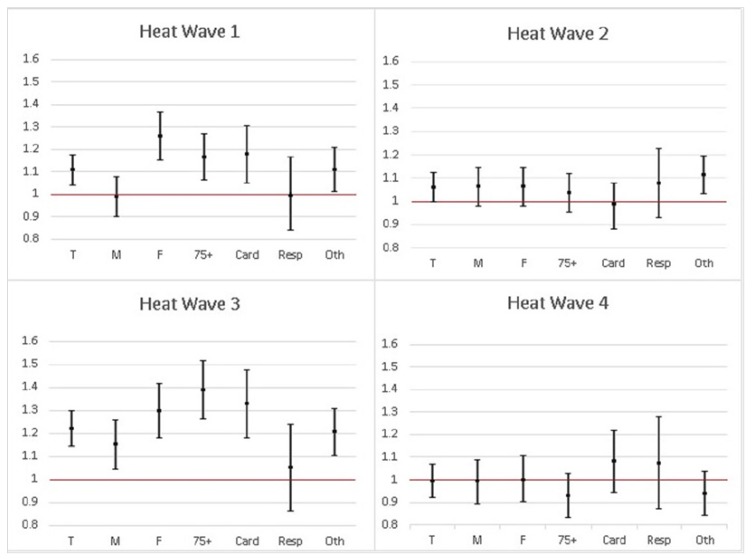
The crude death rates and CI_95%_ of the Extreme Heat Waves between 2013 and 2017 (27 Jul–2 Aug 2015, 5 Aug–14 Aug 2016, 29 Jun–4 Jul 2017, 5 Aug–10 Aug 2017 respectively) by categories. (T: Total, M: Male, F: Female, 75+: 75 years old and above, Card: cardiovascular diseases, Resp: Respiratory diseases, Oth: Others).

Risk ratios for crude, sex-specific, age-specific, and cause-specific deaths with their CI 95% in all episodes are shown in Figure 2. Although the death risk for all deaths were significantly increased also in Heat Wave 2 in 2016, increased death risk in Heat Waves 1 and 3 in 2015 and 2017 were stronger for all deaths, female deaths, elderly deaths, and deaths due to cardiovascular diseases.

## 4. Discussion

This study showed that three heat wave episodes in Istanbul in 2015, 2016, and 2017 caused an increased risk of mortality: 11%, 6%, and 21%, respectively. The total number of excess deaths during the heat wave episodes for three years was 419. This result is consistent with similar studies in different cities. Two studies about summer heat waves in Vienna and in Switzerland in 2003 showed a 13% and 7% increase in daily mortality, respectively [25,26]. On the other hand, the risk ratio is lower than those reported in some other studies, such as a 55% increase for France in 2003 [27], 38% increase for Belgrade in 2013 [28], and 33% for Montreal in 2010 [29]. Various results may be due in part to the duration of the heatwave and magnitude of extreme heat, as well as to the age structure, prevalence of chronic diseases, socio-economic deprivation, the level of emergency preparedness, access to air conditioning, and other factors [30,31]. Some analyses were done to try to characterize some risk factors that could be amenable to preventative public health interventions; below are discussed the importance of the characteristics of the heat wave, age, and categorical cause of death. 

In an epidemiological study about the relation of heat and mortality in London between 1976 and 1996, Hajat et al. [32] found out that the “heat episodes of long duration and of highest temperature have the largest mortality effect.” For example, in the Belgrade study in which a high increase in mortality was found, the duration of the heat wave was nine days and mean daily temperature was 6.7–13.1 °C higher than normal [28]. In this study, the longest heat wave lasted seven days and the mean daily temperatures during the heat wave episodes were 2.3–4 °C higher than the monthly normal [33].

These results seem to show that women are more vulnerable to heat waves than men, but RRs are significantly different only for wave 1. Many other studies show increased mortality in women during heat waves, possibly because women are less heat tolerant than men due to different thermoregulatory and physiological mechanisms [26,27,34,35,36].

Although younger compared to Turkey’s age distribution in general, Istanbul has an aging population. People over 65 constituted 6.7% of the population of Istanbul in 2018 and 8.8% of the total population in Turkey. The ratio was 5.2% in 2008 and is expected to be more than 7% after 2020 [37]. Elderly people older than 75 were more vulnerable to heat waves: the death risk for the elderly population increased by 16% in 2015 and 38% in 2017. Death risks were not significantly increased in other age groups. This result is consistent with many other studies where the association of mortality and heat waves is much stronger for people older than 75 years [27,28,38,39,40,41].

Cardiovascular deaths seemed related to heat waves in Istanbul, while other cause-specific death rates, including those due to respiratory diseases, did not increase. The death risk for cardiovascular disease increased 17% in the 2015 heat wave and 32% in the 2017 heat wave, both higher than the increase of crude death rates. This result is also consistent with previous studies that found that while cardiovascular deaths were related to heat, especially for the elderly [42,43], heat does not seem to have an effect on respiratory mortality [44,45]. It could be related in part to air pollution levels during the heat waves. This study did not take the impact of air pollution into account, although ozone pollution may have an impact on the results as seen in Brisbane during the 2004 heat wave. Thus, this topic needs further research [46]. 

Although the death risk was increased in three (out of four) heat waves in Istanbul, the increase in the 2016 heat wave was not as high as the other two heat waves in 2015 and 2017. The highest risk ratio was for Heat Wave 3 between 29 June and 1 July 2017, i.e., early in the summer, although with a shorter duration. This may be caused by the period of time necessary for adaptation to heat, because people are less tolerant to extreme temperature during the first weeks of summer than the late summer [47], similar to the results in a study of heat waves in London [32]. Additionally, the temperatures during Heat Wave 1 and 3 in 2015 and 2017, respectively, were 4 °C higher than the monthly normals, while in Heat Wave 2 in 2016 the temperature was only 2.3 °C hotter than normal. The second heat wave in 2017 did not increase death rates for the general population or the elderly. This may be seen as an example of the harvesting effect: although the first heat wave in 2017 increased the mortality rate particularly for the elderly, no statistically significant mortality displacement was found. On the other hand, because of the first heat wave in June, and thanks to the warning reports by the meteorological services and media [48,49], people could have taken their own precautions, including moving out of the city, taking advantage of the August holiday season.

Some limitations of this study need explanation. There are two general methodological approaches for heat (or heat wave) and mortality studies. In time series studies, the impact of temperature on mortality is assessed in a defined geographic region over a substantial time period. In heat wave studies, relative risk of death rates in defined heat wave episodes and control episodes (i.e., several days before or after the heat wave in the same year or the similar period of previous years) are examined. An additional approach may be possible if deaths due to heat (or heatstroke) are explicitly recorded and provided as a cause of death, although this kind of data usually underestimates heat-related deaths [50,51]. In this study, the design was imposed by the data available. Although mortality data has been collected and recorded by the health authorities in Turkey for a long time, the daily number of deaths and their categorization according to basic demographics and cause of death have become available only quite recently. Therefore, the long-term trends in daily mortality data that are necessary for time series studies could not be assessed, even for crude death rates. This type of study will only be possible in the future after a sufficient amount of time has passed for time series studies. It is also important to note that heat stress or heatstroke are not recorded in the death certificates that are made available by the regional health directorates. 

Another type of limitation is linked to available data, which has been collected for only a short period on a daily basis, and the lack of information on several potential confounders. As in many such ecological studies, residual confounding is certainly present but could not be estimated properly. Future improvements on data availability for longer periods on variables such as socio-economic levels, quality of dwellings, intra-urban heat islands, and the like would allow a better estimation of risks levels and improve decision-making for population health measures.

Several meteorological indicators may be used in heat wave studies, including mean temperature, apparent temperature, and heat index [23]. Daily minimum temperature is also important because of the recovery effect of low nighttime temperatures [25]. The simple average of the daily mean temperature measured by all meteorological stations in Istanbul was used here, because many factors possibly contributing to mortality, such as location and circumstances of deaths, time of the day, suddenly or after a being hospitalized, etc., could not be distinguished. Furthermore, Istanbul is a big metropolitan city, and the hospitals where the deaths took place and were recorded may be in different districts of the city than the locations in which the onset of the heatstroke, cardiac arrest, or any other events leading directly to death occurred. In addition, using the simple average of all meteorological stations may smooth the extreme measurements of temperatures in the city center, because some of the stations are close to the forested and rural areas of the city.

The definition of a heat wave period is important for the study design. Researchers may choose one of the definitions, such as taking a certain temperature threshold, which is defined by considering the city’s climate normal in comparison to the days with a temperature exceeding a certain percentile (e.g., 90th, 95th, 99th, etc.). Additionally, the extreme heat event must last more than two or three days [52,53]. Defining a warning threshold for a city or region by looking at which extreme heat events make excess death significant is important for public health interventions [54,55]. The heat wave definition in this study (temperatures above the 95th percentile sustained for more than three days) showed a significant relationship between heat events and mortality. However, Istanbul and other cities in Turkey do not have any heat wave warning system or specific local thresholds for determining a heat wave. Similar research projects in other regions of Turkey would be useful for creating a local definition of a heat wave that can highlight overmortality risks.

## 5. Conclusions

This study showed that three heat wave episodes in Istanbul in 2015, 2016, and 2017 caused an increased risk of all-cause mortality: 11%, 6%, and 21%, respectively, without any harvesting effect. The total number of excess deaths during the heat wave episodes for three years was 419. These results are in line with similar studies worldwide in comparable situations for amplitude of effect, age groups most affected, and the importance of cardiovascular deaths in the episodes. Some limitations due to data availability and quality highlight potential future improvements.

Since there are very limited studies about heat waves and mortality in Istanbul related to the past heat waves such as the ones in 2000, 2003, 2007, and 2010, it could not be assessed whether there were any improvement over time in terms of adaptation or behavioral change, as no historic reliable data is available. Since there are no public health intervention plans for heat waves at the local or national levels, further studies about increased death risks due to heat waves in Istanbul and other cities, as well as intervention studies, are necessary in the light of the impacts presented in this study. Other studies on heat-related ambulatory and hospital morbidity could be of great interest too for Istanbul and other regions of Turkey.

## Figures and Tables

**Table 1:** Descriptive statistics of temperatures and deaths by years (for June–August).

Characteristic	2013	2014	2015	2016	2017
Average of Daily Mean Temperatures (°C) (Min–Max)	23.7 (18.6–26.3)	23.7 (17.5–27.6)	23.4 (18.5–27.9)	24.2 (17.3–27.2)	23.5 (17–29.9)
Number of days with daily average of mean temperatures > 95th percentiles (*n*)	0	5	5	7	6
Number of extreme heat waves (*n*)(days)	0	0	1(4)	1(7)	2(3; 3)
Population (*n*)	14,160,467	14,377,018	14,657,434	14,804,116	15,029,231
Daily average number of deaths (*n*) (Rates/100k)	140.0 (0.99)	153.2 (1.07)	157.7 (1.08)	161.9 (1.09)	160.9 (1.07)
Daily average number of deaths by sex (*n*) (Rates/100k)					
Male	75.6 (1.06)	81.5 (1.13)	82.5 (1.12)	86.4 (1.16)	85.8 (1.14)
Female	64.4 (0.91)	71.7 (1.00)	75.2 (1.03)	75.5 (1.02)	75.1 (1.00)
Daily average number of deaths by age groups (*n*) (Rates/100k)					
0–14	9.6 (0.29)	10.7 (0.33)	10.1 (0.31)	10.1 (0.31)	9.7 (0.29)
15–64	38.8 (0.39)	41.3 (0.40)	41.9 (0.40)	42.9 (0.40)	42.6 (0.40)
65–74	28 (3.38)	30.7 (3.62)	31.5 (3.45)	32.8 (3.49)	32.8 (3.31)
≥75	63.5 (12.42)	70.6 (13.36)	74.2 (12.91)	76.1 (12.78)	75.8 (12.08)

**Table 2:** Crude Death Rates and Risk Ratios (RR) of the Extreme Heat Waves between 2013 and 2017 (* including 3 days lag, ** excluding 3 days lag).

Extreme Heat Waves	Start Date	End Date *	Number of Days *	Average of Daily Mean Temp (°C) **	Population	Number of Deaths	Death Rates (x100k)	RR (95% CI)	*p* Value
Heat Wave 1	27 Jul 15	2 Aug 15	7	27.57	14,657,434	1203	1.17	1.11 (1.04–1.18)	0.002
Reference 2013	29 Jul 13	04 Aug 13	7		14,160,467	943			
Reference 2014	28 Jul 14	03 Aug 14	7		14,377,018	1057			
Reference 2016	25 Jul 16	31 Jul 16	7		14,804,116	1198			
Reference 2017	24 Jul 17	30 Jul 17	7		15,029,231	1125			
Reference Total			28	25.64	58,370,832	4323	1.06		
Heat Wave 2	5 Aug 16	14 Aug 16	10	26.97	14,804,116	1658	1.12	1.06 (1.00–1.12)	0.037
Reference 2013	02 Aug 13	11 Aug 13	10		14,160,467	1408			
Reference 2014	08 Aug 14	17 Aug 14	10		14,377,018	1546			
Reference 2015	07 Aug 15	16 Aug 15	10		14,657,434	1603			
Reference Total			30	25.29	43,194,919	4557	1.05		
Heat Wave 3	29 Jun 17	4 Jul 17	6	28.72	15,029,231	1164	1.29	1.21 (1.14–1.30)	<0.001
Reference 2013	27 Jun 13	02 Jul 13	6		14,160,467	839			
Reference 2014	26 Jun 14	01 Jul 14	6		14,377,018	930			
Reference 2015	02 Jul 15	07 Jul 15	6		14,657,434	977			
Reference 2016	30 Jun 16	05 Jul 16	6		14,804,116	955			
Reference Total			24	23.78	57,999,035	3701	1.06		
Heat Wave 4	5 Aug 17	10 Aug 17	6	27.09	15,029,231	926	1.03	0.99 (0.92–1.07)	0.847
Reference 2013	03 Aug 13	08 Aug 13	6		14,160,467	820			
Reference 2014	02 Aug 14	07 Aug 14	6		14,377,018	911			
Reference 2015	08 Aug 15	13 Aug 15	6		14,657,434	950			
Reference Total			18	25.27	43,194,919	2681	1.03		

**Table 3:** Sex-specific Death Rates and Risk Ratios of the Extreme Heat Waves between 2013 and 2017.

Heat Wave	Men					Women				
	Population	Number of Deaths	Death Rate (x100k)	RR (95% CI)	*p* Value	Population	Number of Deaths	Death Rate (x100k)	RR (95% CI)	*p* Value
Heat Wave 1	7,360,499	572	1.11			7,296,935	631	1.24		
Reference Total	29,290,760	2315	1.13	0.98 (0.90–1.08)	0.718	29,080,072	2008	0.99	1.25 (1.15–1.37)	<0.001
Heat Wave 2	7,424,390	863	1.16			7,379,726	795	1.08		
Reference Total	21,697,378	2374	1.09	1.06 (0.98–1.15)	0.128	21,497,541	2183	1.02	1.06 (0.98–1.15)	0.154
Heat Wave 3	7,529,491	585	1.29			7,499,740	579	1.29		
Reference Total	29,121,768	1977	1.13	1.14 (1.04–1.26)	0.004	28,877,267	1724	1.00	1.29 (1.18–1.42)	<0.001
Heat Wave 4	7,529,491	478	1.06			7,499,740	448	1.00		
Reference Total	21,697,378	1396	1.07	0.99 (0.89–1.09)	0.801	21,497,541	1285	1.00	1.00 (0.90–1.11)	0.991

**Table 4:** Age-specific Death Rates and Risk Ratios of the Extreme Heat Waves between 2013 and 2017.

Heat Wave	0–14					≥75				
	Population	Number of Deaths	Death Rate (x100k)	RR (95% CI)	*p* Value	Population	Number of Deaths	Death Rate (x100k)	RR (95% CI)	*p* Value
Heat Wave 1	3,302,582	69	0.30	1.05 (0.80–1.36)	0.740	337,192	600	25.42	1.16 (1.06–1.27)	0.002
Reference Total	13,165,698	263	0.29			1,345,706	2067	21.94		
Heat Wave 2	3,301,723	98	0.30	1.08 (0.86–1.36)	0.524	345,017	787	22.81	1.03 (0.95–1.12)	0.507
Reference Total	9,842,118	271	0.28			975,254	2164	22.19		
Heat Wave 3	3,324,439	49	0.25	0.75 (0.55–1.01)	0.059	362,627	647	29.74	1.38 (1.26–1.52)	<0.001
Reference Total	13,143,841	260	0.33			1,320,271	1702	21.49		
Heat Wave 4	3,324,439	52	0.26	0.91 (0.67–1.24)	0.556	362,627	443	20.36	0.93 (0.83–1.03)	0.179
Reference Total	9,842,118	169	0.29			975,254	1283	21.93		

**Table 5:** Cause-specific Death Rates and Risk Ratios of the Extreme Heat Waves between 2013 and 2017.

Heat Wave	Cardiovascular				Respiratory				Others			
	Number of Deaths	Death Rate (x100k)	RR (95% CI)	*p* Value	Number of Deaths	Death Rate (x100k)	RR (95% CI)	*p* Value	Number of Deaths	Death Rate (x100k)	RR (95% CI)	*p* Value
Heat Wave 1	400	0.39	1.17 (1.05–1.31)	0.005	172	0.17	0.99 (0.84–1.17)	0.918	631	0.61	1.11 (1.01–1.21)	0.025
Reference Total	1360	0.33			691	0.17			2272	0.56		
Heat Wave 2	492	0.33	0.98 (0.88–1.08)	0.649	265	0.18	1.07 (0.93–1.23)	0.350	901	0.61	1.11 (1.03–1.20)	0.007
Reference Total	1470	0.34			723	0.17			2364	0.55		
Heat Wave 3	406	0.45	1.32 (1.18–1.48)	<0.001	145	0.16	1.03 (0.86–1.24)	0.718	613	0.68	1.20 (1.10–1.31)	<0.001
Reference Total	1188	0.34			541	0.16			1972	0.57		
Heat Wave 4	305	0.34	1.07 (0.94–1.22)	0.320	155	0.17	1.04 (0.87–1.25)	0.669	466	0.52	0.93 (0.84–1.04)	0.205
Reference Total	820	0.32			428	0.17			1433	0.55		
